# Achieving Behavioural Advancement through the Dynamic Maturation Model and the Assessment of Parent–Child Interactions in a Music Therapy Context

**DOI:** 10.3390/bs12090338

**Published:** 2022-09-15

**Authors:** Rachel Swanick, Efthymios Papatzikis

**Affiliations:** 1 Chroma, Herefordshire, Ross-on-Wye HR9 7US, UK; 2 Department of Care Sciences, University of South Wales, Pontypridd CF37 1DL, Wales, UK; 3 Department of Early Childhood Education and Care, Oslo Metropolitan University, 0167 Oslo, Norway

**Keywords:** attachment, parenting, assessment of parent–child interaction, dynamic maturational model, music therapy, assessment

## Abstract

Using concise and valid assessment tools before embarking on therapy with clients not only provides key information on areas of concern, but it can also focus the ongoing therapy, giving a greater chance of positive outcomes. This article discusses the use of the Dynamic Maturational Model (DMM) and the Assessment of Parent–Child Interactions (APCI) as a framework for assessment with adoptive families in a music therapy service. It is proposed that using a Screening Family Formulation (SFF) as detailed through the DMM alongside the APCI can provide this secure foundation for assessment. It is proposed that the DMM promotes the use of the professional–parent relationships as a useful outline for the parent–child relationship alongside a thorough understanding of the issues faced by the family. In addition, the APCI enhances the assessment process by providing clinical and therapeutic evidence of areas of development for the family.

## 1. Introduction

A good assessment for a family can lead to good treatment plans and, subsequently, good outcomes for the family. Quinlivan et al. [1] found that clients who have access to psychosocial assessments at the time of referral feel more supported by services and are more likely to keep themselves safe. Furthermore, the research team found that appearances and perceived capacity of the clients were influenced when they were offered an assessment, possibly implying that when clients appear to be coping well, they may not have their needs taken seriously [1]. The Social Care Institute for Excellence describes good assessments as a ‘systematic set of ideas’ that can be used to provide a way to collect and understand information. Therefore, an assessment at its best “would be underpinned by understandings of human experience and action, offer explanation of the situation being assessed and how to respond, and be supported by compatible models and tools for conducting the assessment” [2]. In this framework, the best combination of tools in terms of content and validity, as well as training on these, should be highly considered.

First of all, having a robust assessment tool for a therapeutic service means investment from therapists. Assessing therapists can be seen as facilitators with a view to empowering the family to succeed. Training can be expensive and take many months or years to feel stable with the role of assessor. However, when treatment is started, it should be ensured that the best possible opportunities for relevant goals are given to the family. Staff and therapists will need to be trained and confident in their work, as well as having the ability to remain objective towards the family, i.e., not falling into the need of ‘fixing’ the family or providing advice. Therapists must be able to ‘let the assessment happen’ without intervening with the family—within reason if safety is an issue. Watching a mother, for example, struggling to engage her baby or put a boundary in for a chaotic 9-year-old can be difficult for a therapist to observe when one of the main reasons to become a therapist is help and nurture. Training to be an assessing therapist therefore can upset working practices and some therapists might struggle with the idea of observing rather than interjecting, wondering how they keep their authentic selves in those moments. It is at these times that assessing therapists need to feel comfortable in their techniques or protocols so that their unique vocational skills of compassion and hope can be used. Using compassionate thought to communicate with a family in distress may build a more trusting, positive, and hopeful alliance; especially useful when assessments are short by nature. Joyce, et al. [3] found Yalom’s idea of the Installation of Hope was a ‘global factor’ when trying to understand the potential positive impact of therapy. When feeling hopeful, clients are more likely to believe in their future and value, and this is then linked to self-esteem, positive relationships, and overall wellbeing [4,5].

Furthermore, assessments can prove difficult for professionals and services to provide, as they may find them expensive, time consuming, and anxiety provoking for the families [6,7]. Therefore, employing assessment tools that are effective, reasonably priced whilst covering nonverbal communication, attunement, and information processing adds to the challenge of providing effective treatment plans for families. Based on this, Colegrove and Havivhurst have specifically commented on the lack of interventional and assessment tools in parent–child dyads, even though there is plenty of evidence suggesting that this is key to understanding family dynamics [8]. Finally, when offering assessments to families, it is important for the tools used to be empirically validated. For a tool to be validated, it must be able to measure what it claims to measure and concentrate solely on that. Ali et al. [9] found that the ‘gold standard’ of assessment and screening tools were those conducted by a trained professional, conducted, and used in a multiple of settings and developed for specific diagnostic populations.

In light of the above and considering the need to effectively provide concise and valid assessment tools for us to not only collect key information on areas of concern, but also to focus on the ongoing therapy with a greater chance of positive outcomes, we propose and discuss in this article the use of the Dynamic Maturational Model (DMM) in combination with the Assessment of Parent–Child Interactions (APCI) as a pioneering framework for assessment with adoptive families. On the one hand, we consider the Dynamic Maturational Model (DMM) as it is based on established yet ground-breaking work of developmental psychology. Ainsworth’s Strange Situation assessment [10], which is part of the DMM, has been effectively and longitudinally used around the world to understand attachment behaviors in families. In this model, Ainsworth sets out the four main types of attachment behaviors, which are used in a vast rage of psychological settings. On the other hand, we consider the Assessment of Parent–Child Interactions (APCI) because of its uniqueness. It is the only music therapy assessment tool which aims to understand parenting capacity in families who are experiencing challenged attachment strategies [11]. 

## 2. The Dynamic Maturational Model 

The Dynamic Maturational Model (DMM) was created by Patricia Crittenden and is a strengths-based model of development that emphasizes the dynamic interaction of maturation across the lifespan. It is an empirically tested model, used across the world, and aims to think about attachment strategies through the lens of our needs for safety and comfort [6]. The DMM poses that human behaviors are based on the need for survival: we do what we can to stay out of danger and to ensure that we can reproduce our genetic line for the future.

**Figure 1:**
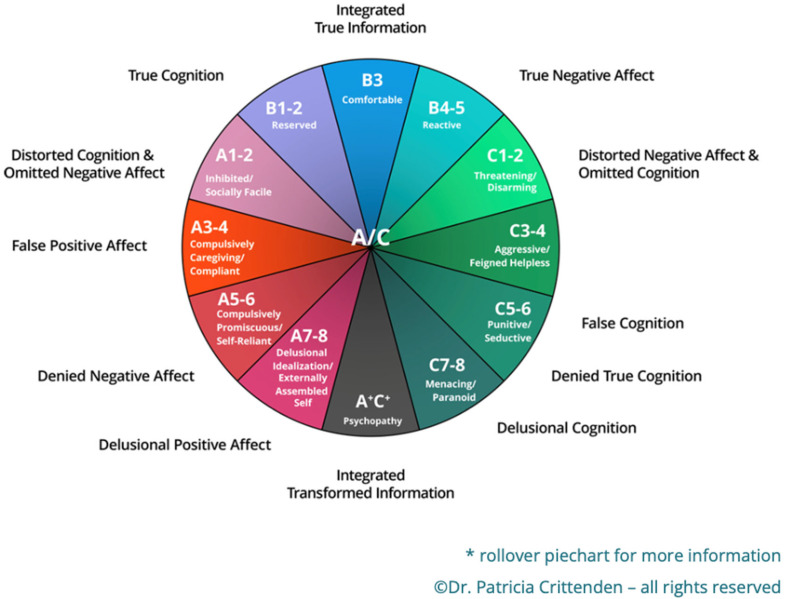
The DMM Model, www.familyrelationsinstitute.org (accessed on 15 June 2022). Permission granted.

In the DMM, the word ‘strategy’ replaces the term ‘behaviors’ when thinking about attachment. This subtle shift to strategy suggests a dynamic action where individuals have a neurological blueprint for ways of gaining attention from attachment figures [6]. It also suggests that attachment feelings are flexible and that individuals use a range of strategies within their different attachment relationships (parental, peer, spousal) in order to get what they need. Most importantly, the DMM poses a new set of attachment strategy descriptors, which have been finely tuned to add to the Ainsworth and Bowbly theories [12,13] through many years of investigation (see Figure 1).

## 3. The Assessment of Parent–Child Interaction (APCI)

The APCI is an empirically tested and validated assessment tool which uses musical interactions as a metaphor for the dynamics being played out in the family [14]. The assessment is used for children aged between 4 and 12, although other ages are considered. The APCI aims to uncover the nonverbal patterns in the family relationship, providing clear and concise information on attachment behaviors and clinical parenting skills. In particular, the APCI was specifically created for the field of child protection and is unique in its use of music. Using music to think about the family dynamics makes it a highly relevant tool to capture the objective elements of the parent child dynamics. In music therapy literature, much has been written about the musical qualities of communication and the impact of this on the attunement between family members [7,15,16,17]. Jacobsen et al. [14] found that music therapy significantly improved nonverbal communication and attunement in family dynamics in as little as ten sessions.

The APCI is a strength-based tool and the motto for all assessing therapists is to give the family the “Benefit of the Doubt” [7]. This means that alongside uncovering and acknowledging the difficulties the family are facing, space must also be given for what is working well and successfully in the family. Even though the APCI consists of two, time-limited sessions, families have frequently stated that they feel seen and heard by the assessing therapist and that it has been useful when thinking about how the family interact with one another [7]. 

The APCI is undertaken by a trained therapist who leads two-planned assessments sessions, held one week apart and video recorded. The videos of the interactions between the parent and child are analyzed using Event Based Analysis [18], with notes taken of the quantitative events and the qualitative details. An in-depth report is then provided for the family which gives information on the attachment behaviors of the dyad and the parenting capacity for attunement and understanding. The attachment behaviors described through the APCI are in line with the Ainsworth model of attachment [19]. 

Finally, the APCI consists of four activities, which are a mixture of structured and unstructured. The parent–child dyad is encouraged to follow and lead each other in various ways, without using words and relying on musical interactions and nonverbal communication such as body and facial gestures. In supporting families, the parent will give the child a sense of autonomy and freedom when exploring the musical sounds and activities. In challenged families, the balance of autonomy and boundaries will be difficult for the parent and child and there may be times when both family members are not attuned to each other’s needs. When a child has good enough parenting, they expect bad situations to be recovered with support from the parent. However, with positive attunement happening around 30% of the time [20], repairs and reparation of mis-attunement become imperative, both to the well-being of the child and family and for positive outcomes in therapy [21]. Supporting parents to build reflective capacity around their own behaviors, alongside the child’s behaviors, becomes a significant part of the therapeutic process [22], and this can be encouraged through the APCI and subsequent therapeutic recommendations after the assessment.

## 4. The Effectiveness of DMM

The DMM posits that the presenting issues at referral are often *not* the root of the issue in the family. That is, the negative feelings and worries have been displaced into the client and they are holding these feelings for the whole family. By looking for the discrepancies in behaviors through nonverbal gestures and acknowledging what it feels like to be in the room with the family through attunement, the therapist can begin to undercover the unsaid experiences of the family. Often, the first line of assessment when using the DMM is for the therapist to undertake a Screening Family Formulation (SFF) with the family. The SFF uses tested techniques such as Maslow’s Hierarchy of Needs [23] and the Adverse Childhood Experiences [24] checklist to begin to understand the family’s psychological position. To realize in more detail where the SFF stands in the DMM delivery process, we will employ here a descriptive example of a case study (for a full descriptive account of this case study, please see Appendix A). In this context, when the Townsend family were referred for assessment, Mr. and Mrs. Townsend were concerned about Joanna, their adopted teenage daughter who had recently self-harmed. When asked if they knew what the source of the anguish was for their daughter, they stated that they ‘did not know’. This statement of not knowing from Mr. and Mrs. Townsend became a key element of the SFF. However, as the exploration of the case progressed, there were many indicators of Joanna’s distress that her parents had not been able to acknowledge. Joanna seemed unable to express her feelings out loud (to her parents or another person) and she was showing those emotions through self-harm. Joanna had also been hiding personal items away from her parents and this could be an indicator of her having shame around her own needs and more negative feelings. In contrast to Joanna’s outward self-harm and overdose, her parents were worried about what other people would think about their family and they were inhibiting negative feelings by focusing on ‘doing’ or behavioural issues with Joanna. To effectively approach the above concerns, and in line with the DMM model, the assessing therapist started first issuing questions around danger and safety to further understand the strategies of the family: *where was the danger at this point and what was making Joanna feel unsafe in the here and now*?

Understanding the family in relation to an adapted version of Maslow’s Hierarchy of Need opened up new thinking around the source of their challenges. Firstly, there was a discrepancy in the level of needs the parents and their daughter were feeling. According to Crittenden [6], discrepancies give us more information on the issues faced by families as the hidden information often represents unspeakable feelings the family are facing. Joanna’s parents had a sense of comfort from each other, whilst Joanna was outside of this and possibly feeling unprotected by them. Furthermore, with Joanna in an experimental stage of life where she was exploring who she was in a deep and age-appropriate way, having a protector attachment figure to return to may have felt imperative to her wellbeing. Because of all these assessment realizations, the exploratory assessment questions started becoming more focused on Joanna: *Who am I*? *Where do I belong*? *Will I be rejected*?

However, instead of the Screening Family Formulation (SFF) focusing solely on the need for safety in the face of an unknown danger for Joanna, the assessing questions also started approaching the family as an undivided system as the screening went along. They were covering wider topics, although failing to get to the critical issue of the family’s problem. It was only when the family’s feelings around sexuality began to be explored—key not only with this referral but also a pivotal issue in the DMM in the need for humans to reproduce—that more important information started to come to the surface. When asked, Mr. and Mrs. Townsend found it extremely difficult to talk about the reasons they adopted, possible issues around infertility and reproduction, and what it might feel like for Joanna to reproduce and not create a genetic heir to their family. Farnfield [25] stated that around 20% of adoptive parents have unresolved loss and trauma. This can impact negatively on the child more than insecure attachment strategies in parenting. Furthermore, given Joanna’s adolescent age, she was beginning to explore her own sexuality through fluidity and possible relationships, which again, according to Farnfield [25], could have been triggering trauma responses in her parents because of their unresolved loss. 

In this case of the DMM delivery, as in other similar assessment cases, the therapist may feel by the end of the SFF, that more questions may arise than answered. Hence, neither the family nor the therapist may be able to articulate where critical causes lay. This is where a tool like the APCI may come into play to help the family system explore non-verbal ways to communicate as a unified core. 

## 5. The Effectiveness of the APCI

To start to understand the underlying communication styles the Assessment of Parent–Child Interactions [7] can provide, a full delivery needs to be undertaken so as to assess attachment behaviors and parenting capacity. Once more, to exemplify its delivery mode in a real setting, the same case study will be briefly approached as before, with more details found in Appendix A. Analysis of the interactions in the music therapy sessions showed that for Joanna and Mr. Townsend, their mutual attunement, nonverbal communication, and emotional parental responses were in the normal ranges. Joanna seemed to be more playful with Mr. Townsend than with Mrs. Townsend, although there was a sense that Mr. Townsend was mindful of Joanna’s physical vulnerability, which effected the level of playfulness and enjoyment in the session. For example, during the follow my leader activity, Joanna showed high energy levels and dynamic music playing. Mr. Townsend met her gaze and facial expressions well and although he started to meet Joanna’s musical energy too, he quickly diminished his efforts, in the possible hope that Joanna would follow and not become over excited. 

Although, it must be noted that Mr. Townsend’s score for emotional parental response was in the normal range, it was still a low score. Both parents were dependent on support from the assessing therapist, which could indicate a feeling of uncomfortableness in the assessment sessions. Furthermore, the family seemed more comfortable in the structured activities, rather than the unstructured ones, again highlighting a need for supporting boundaries within the family. The themes being uncovered through the SFF were now clarified with the addition of the APCI, showing exactly where Joanna’s overt emotional requests were either ignored or thought of as too overwhelming. 

## 6. The Combined Assessment Value

One of the reasons a family may be in crisis is that the parents have transferred their experiences and feelings of past relationships on to the child, leading to the parent not always being able to respond to the needs of the child and a ‘mismatch’ in communication between parent and child being caused. Furthermore, with the parents dealing with three levels of challenges—micro (the person), meso (the family), and the macro (societal) [26]—thinking about the family in their individual context becomes imperative. As the DMM provides the information basis to work on, the APCI aims to understand the nonverbal communication of the family, and also holds the ideals of the importance of family work (rather than purely concentrated on one person). Therefore, the use of both tools in combination can be very helpful for professionals working with families in crisis. 

Both the APCI and the DMM use the theories of attunement and the importance of non-verbal communication when thinking about family dynamics. In this context, Stern [27] discussed the special attunement of mother–infant interactions and how the ordinary mother responds to the baby through voice, body gestures, facial expressions, and rhythm. When the mother (or main caregiver) has a good level parenting capacity and is attuned to the child, the mother matches and confirms the feelings and reactions of the infant, creating a sense of safety and comfort. Knapp and Hall [28] described the importance of being able to encode and decode signals of nonverbal communication (that creates signals as well as receiving and understanding them) when building and maintaining relationships with others. Stern, Knapp, and Hall propose that the key time for this skill to be embedded is in early childhood and requires emotionally available parents. Tuomi [29] has discussed the subject of families in music therapy and the importance of including parents in the work with children in order to increase a sense of bonding within the family, while Pasiali [30] has written extensively on the importance of building emotional resilience in families as a way to increase a high sense of life satisfaction.

To think about the attunement and nonverbal communication as set out in the APCI and DMM combined, a musical metaphor would be for the therapist to listen to the harmonies around the child. For example, are the family working together, what are the rhythms of each family members, their tempi, dynamics, melodies, rests, and silences? Then, through clear and consistent communication, the therapist can model how to talk about the unsaid things, the difficult feelings, the challenges, and the successes of the family so that they are teaching the parents how to be with their own children in a more positive way. The aim of the therapeutic work becomes trying to create empathetic parent–child relationships, and this is echoed in both the APCI and the DMM [7,22]. 

## 7. Discussion

With the Dynamic Maturational Model offering the theory that the reason for referral is often not the root of the family’s concerns, professionals need to approach the referral of child into their service with curiosity, compassion, and hope. Most often, professionals will try to change the child’s behavior through therapeutic intervention and parents will not be included or thought about during that time. Over the long term, though, this will not have a great impact. However, if professionals are interested and thoughtful with parents, they can help parents to identify and understand dangers more accurately whilst helping them to respond to their child in a way that encourages attachment and feelings of safety. 

If the parent’s attachment strategies are changed, this will elicit rapid and long-lasting change for the family, creating more security and stability for all [31,32]. This idea becomes a sticking point in many services as it is mainly the children being referred who received funding for therapeutic work. Surely, if the best chance of positive change is by working with parents, services and funders need to look at the family as whole and not the child as an island. “*Change the danger, not the child*” [33].

This article highlights a preliminary link to support change for families through the use of the DMM and the APCI at the start of the family’s therapeutic journey. There is a need for consistent assessment or screening where professionals are open to the underlying reasons for referral of the family. Enabling professionals with the confidence to be curious about the family’s experiences may create a sense of safety and hope for their future together. The following areas of thought and development for services working with trauma and families are proposed, based on the initial and positive experiences presented here:

**Assessment:** Good assessment leads to good treatment which leads to good outcomes for the family. Assessment must include all family members so that a full understanding of the levels of safety and comfort in the home is given. Spend time getting to know the family over a few sessions so that discrepancies can be seen and use the information gained in the first interactions to create safety for all family members.

**Compassion, Curiosity, and Hope**: Managers, commissioners and therapists need compassion and curiosity for clients and each other in their interactions. Holding the hope for families when times are difficult can be a sustaining event for both family and therapist. Professionals asking *why is this behavior happening now and what does it mean*? will help families feel held.

**Be Family Centered:** All family members are part of the problems and the successes, whether they are present or not. Some members may feel elusive, and it is important for professionals to be flexible in their thought process and their time and resources.

**Treatment Plans**: Using assessment information on safety and comfort, therapists can create clear and individual goals for the family, with each member included. Flexibility is key—if it is not working, the treatment is not right. Try again with curiosity and compassion.

**Exclusion and Inclusion:** Having an exclusion as well as an inclusion policy. If the family need a level of support that we are not skilled or prepared to do, we need to refer on to the appropriate place. No treatment is better than wrong treatment.

**Building resilience**: Trauma and family work is emotionally difficult. In trying to create better resilience in parent–child and family relationships, we need to create and promote resilience and wellbeing in staff and professionals. As in the therapeutic work, if we support the parents, the child is better looked after. Therefore, if we support professionals, they are more able to help families.

**Evaluation**: We have assessed, we have included, we have reflected. Now, we need to evaluate—is the therapy working? If not, why not? Once we know, we can make changes so that positive change can happen. 

**Early Intervention**: Equipping families with a sense of support may encourage them to ask for help sooner, i.e., before a crisis takes hold. Giving services resources for good assessment and having enough staff to stay with families for a longer period of time may lessen the need of the families and give them a better chance for a positive life together. 

## Figures and Tables

**Figure A1:**
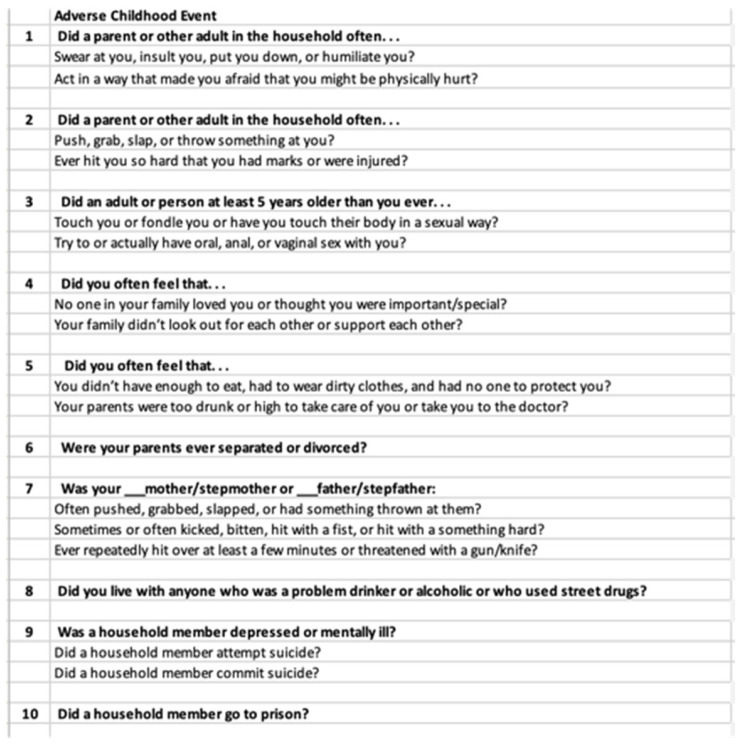
Adverse Childhood Events for the Townsend Family.

**Figure A2:**
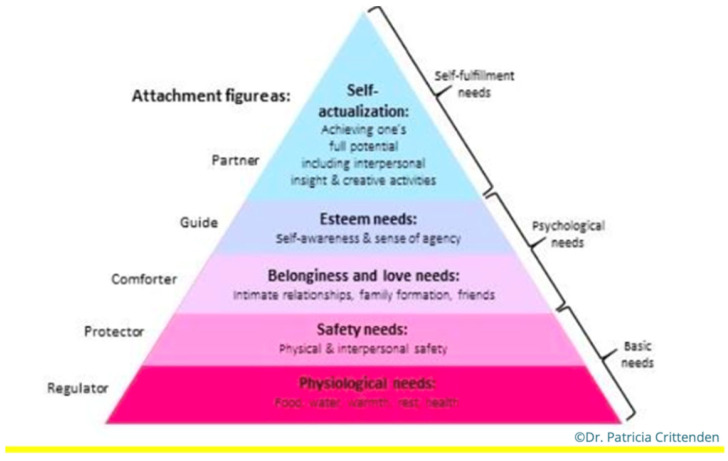
DMM version of Maslow’s Hierarchy of Needs. Permission granted.

**Figure A3:**
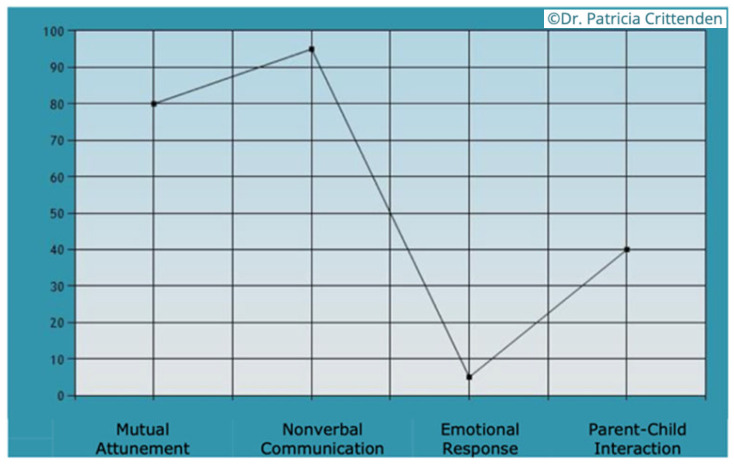
Joanna and Mrs. Townsend.

**Figure A4:**
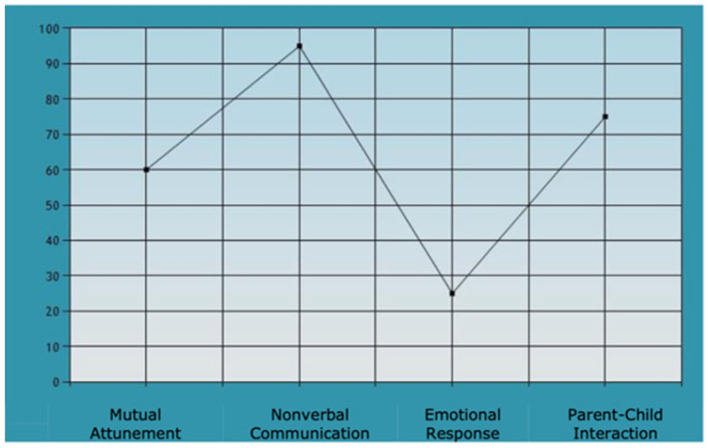
Joanna and Mr. Townsend.

**Figure A5:**
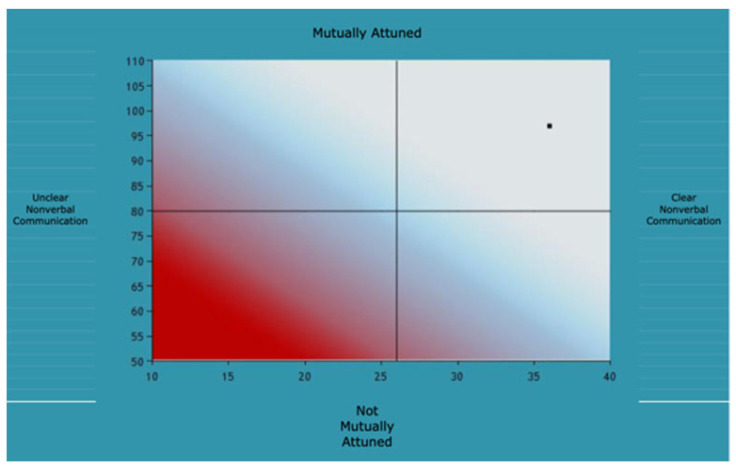
Joanna and Mrs. Townsend.

**Figure A6:**
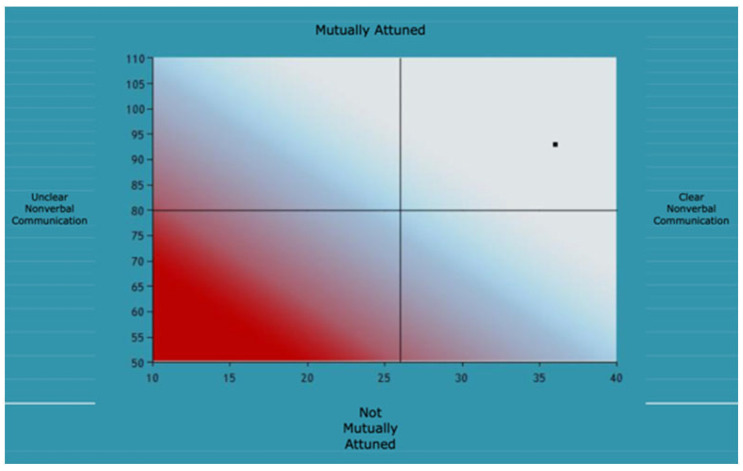
Joanna and Mr. Townsend.

**Figure A7:**
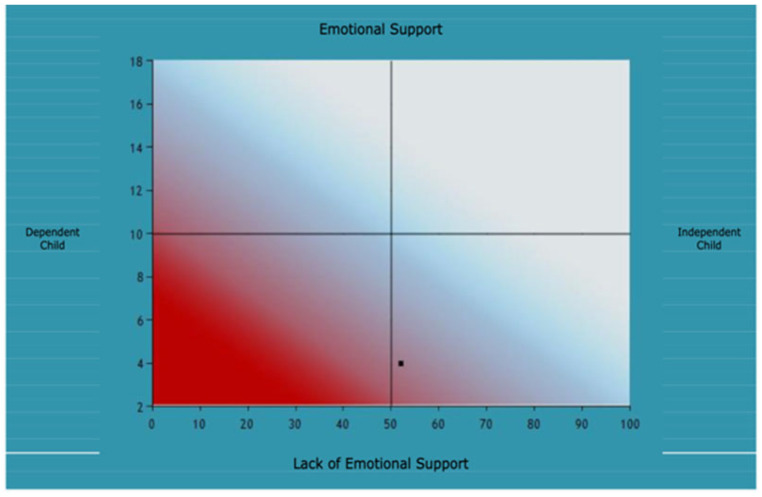
Joanna and Mrs. Townsend.

**Figure A8:**
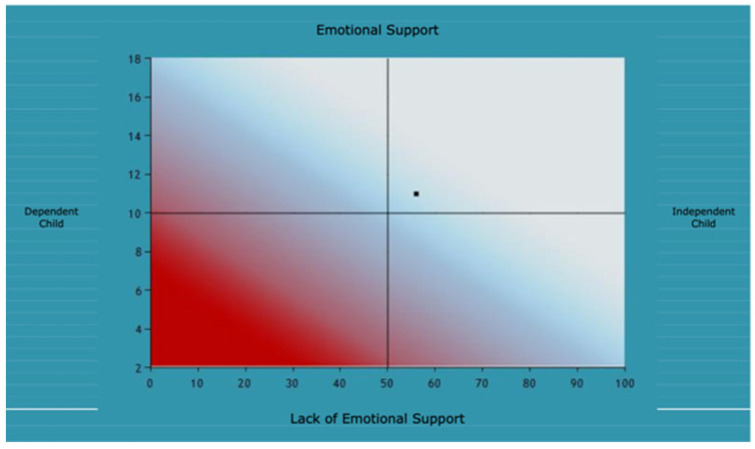
Joanna and Mr. Townsend.

**Figure A9:**
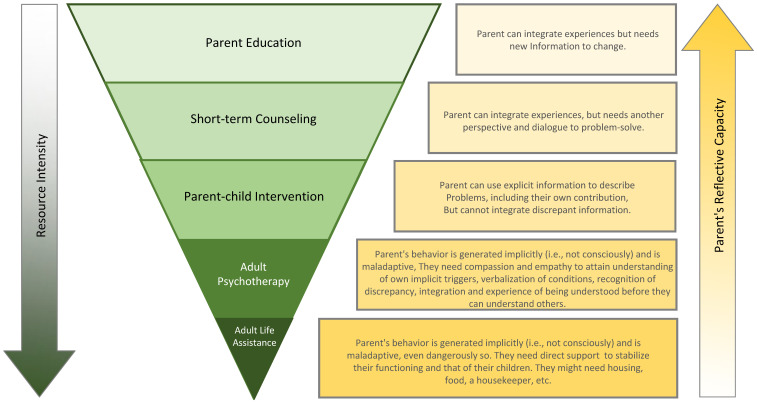
The DMM Gradient of Interventions.

## Data Availability

The data presented in this study are available on request from the corresponding author. The data are not publicly available due to privacy reasons (preservation of anonymity).

## Appendix A. Case Study—Using the DMM and the APCI

Using the DMM as a tool, the following case study describes a Screening Family Formulation (SFF) [1] and an Assessment of Parent–Child Interaction (APCI). The main aim of the SFF is to understand the functions of the behaviors of the family in relation to safety and comfort. The SFF assumes that behavior functions to keep people safe and that, as a strengths-based model, this behavior has been the right thing to do in certain contexts. Therapists leading the work with the family will take this understanding and use it to gently change the way individuals process information of threat so that safety and comfort can be sought when needed. Practically, the SFF contains ten tasks which look at the family holistically, with thought given to the levels of safety each family member feels and needs. The assessing therapist is asked to make a statement on intimacy, for example, in a concise manner. Then, using knowledge gained from the DMM model, the therapist writes a conclusion based on the information gained so far, and suggests questions to further understand the family’s motivations and strategies.

Following the SFF, an APCI was undertaken with the family with the aims of providing quantitative and qualitive information attachment styles and parenting capacity in the family. The APCI, once described by a trainee as “Truthful Kindness”, highlights the positives and challenges of the family through musical metaphor. Using four activities on non-verbal communication, mutual attunement and the emotional parental responses, the assessing therapist can understand patterns of interaction in the family. Therapists are trained to notice the difficulties that are presented in the family and what is working well so that families start their therapeutic journey in way that promotes confidence within the individuals. Both the SFF and the APCI hold hope for the family at the center of the assessments.

**Meeting the Family:** The following case study has been anonymized, although consent was given by the family to take part in the assessment, supervision, and sharing of information for this article.

**Brief History:** Joanna, 15, was referred for a specialist assessment by her adoptive parents. Joanna had been removed from her birth mother at birth, to a safe foster home. She remained there until she was 18 months old when she went to live with Mr. and Mrs. Townsend. In later childhood, Joanna had been assessed for ADHD and ASD, with a slightly raised score on ADHD only.

**Problem at Referral:** Joanna had recently taken an overdose and her parents did not understand why this had happened. Through gentle questioning by the therapist, Mrs. Townsend revealed that Joanna had been in contact with her birth family, and this had brought on some behavioural changes, including the onset of ‘fits’ (physical shaking) and self-harm. Joanna had also started to have questions about her gender and identity.

**Planned Intervention:** To support the family, an APCI was undertaken alongside an SFF. The SFF was undertaken with supervision by an expert in the field as part of a certification program. The SFF described here is abridged for the purposes of this article.

**Intervention Narrative:** In the initial referral, Mr. and Mrs. Townsend stated that they did not understand why Joanna had become distressed. However, as the exploration of the case progressed, there were many indicators of Joanna’s distress that her parents had not been able to acknowledge. Joanna seemed unable to say her feelings out loud (to her parents or another person) and she was showing those around her those emotions through self-harm. Joanna had also been hiding personal items away from her parents and this could be an indicator of her having shame around her own needs and more negative feelings [1]. In contrast to Joanna’s outward self-harm and overdose, her parents were worried about what other people would think about their family and they were inhibiting negative feelings by focusing on ‘doing’ or behavioural issues with Joanna. In line the DMM model, the assessing therapist used questions around danger and safety to further understand the strategies of the family: *where was the danger at this point and what was making Joanna feel unsafe in the here and now*?

Mr. and Mrs. Townsend were asked to complete an Adverse Childhood Event checklist (See Figure A1). Mr. Townsend stated a small incident as a child and Mrs. Townsend, who completed the form, said that she had had no negative experiences as a child and neither had Joanna. The average score for the general population is approximately five adverse experiences. Given that the family had stated such a low score, the assessing therapist felt that this concurred with the inhibited negative affects stated above. The questions from the early part of the work, around “*where is the danger*?”, remained in place when thinking about the family.

Maslow’s Hierarchy of Need (Figure A2) was used to highlight where the family were functioning in relation to their emotional and physical needs. Alongside the traditional levels of needs such as physiological needs, the DMM proposes the type of caregiver the individual may need at each level (Safety Needs with Protector, for instance). With the Townsend family, the parents and daughter seemed to be presenting at different levels. Mr. and Mrs. Townsend had comfort and security in each other and were actively seeking support, therefore, it was felt they were presenting at the ‘Belongingness and Love Needs’ and needing a ‘Comforter’ in their attachment relationships. Joanna, as she was self-harming, questioning her identity through gender fluidity, seeking information on birth family and at an adolescent age, was presenting at the level of ‘Safety Needs’, requiring a ‘Protector’ from her attachment figures.

Understanding the family in relation to the adapted hierarchy opened up new thinking around the source of their challenges. Firstly, there was a discrepancy in the level of needs parents and daughter were feeling. According to Crittenden [6], discrepancies give us more information on the issues faced by families as the hidden information often represents unspeakable feelings the family are facing. Joanna’s parents had a sense of comfort from each other, whilst Joanna was outside of this and possibly feeling unprotected by them. Furthermore, with Joanna in an experimental stage of life where she was exploring who she was in a deep (and age appropriate) way, having a protector attachment figure to return to may have felt imperative to her wellbeing. The exploratory assessment questions became focused on Joanna: *Who am I*? *Where do I belong*? *Will I be rejected*?

Another stage of the SFF asks the family about somatic conditions as a possible link to hidden distress being embodied within the individuals. Mr. and Mrs. Townsend reported no ill health or somatic conditions, whilst there was a heavy focus on Joanna’s ill health. The family were waiting for a diagnosis relating to Joanna’s physical fits and her parents linked the start of these issues to the specific week when Joanna had unpermitted contact with her birth family. With Joanna’s parents not reporting symptoms of somatic distress, the therapist felt there was a reinforcement of the hidden aspect of Mr. and Mrs. Townsend’s emotional world, and possible turmoil. This is in contradiction to Joanna’s many somatic symptoms which include self-harm, taking an overdose and a possible cognitive/physical condition. When assessing the information presented so far, themes of unspeakable feelings and question the identity of the self were presented for both parents and daughter.

So far, the SFF had been focused on the need for safety in the face of an unknow danger for Joanna and the family. Instead of the assessing questions becoming more focused for the family as the screening went along, they were covering wider topics and failing to get to the critical issue of the family’s problem. It was when the family’s feelings around sexuality began to be explored—key not only with this referral but also a pivotal issue in the DMM in the need for humans to reproduce—that more important information started to come to the surface. When asked, Mr. and Mrs. Townsend found it extremely difficult to talk about the reasons they adopted, possible issues around infertility and reproduction, and what it might feel like for Joanna to reproduce and not create a genetic heir to their family. Farnfield [28] stated that around 20% of adoptive parents have unresolved loss and trauma. This can impact negatively on the child more than insecure attachment strategies in parenting. Furthermore, given Joanna’s adolescent age, she was beginning to explore her own sexuality through fluidity and possible relationships, which again according to Farnfield [28] could have been triggering trauma responses in her parents because of their unresolved loss.

Joanna’s parents, perhaps unable to find a way to say the unspeakable distress around these issues, could have been inhibiting them in such a way that there were imperceptible changes in the attachment strategies within the home. Through the SFF, the assessing therapist proposed that the parents were using a Type A 3–4 strategy, the inhibition of negative affect and compulsive caregiving, whilst Joanna was using a Type C 3–4, moving between anger and feigned helplessness. With the difficulties for Joanna’s parents to speak about their adoption journey and feelings of intimacy and reproduction, the already conflicting attachment strategies in the family were moved into a state of ‘disorientation’ [29]. This is when the source of distress is omitted from memory and does not fully understand why situations are happening. With the Townsend family, it may have meant that they were experiencing internal conflict about what to do because information on safety was hidden (omitted) from their conscious. The resulting behaviors are neither ‘self-protective or comfort-eliciting’ in this situation, meaning that no one in the family has their needs met. With Joanna exploring her sexuality in an overt way (and in line with her proposed Type C attachment strategy), her behaviors were in counterpoint to the inhibition of sexuality displayed by her parents. At her age, Joanna had the potential to reproduce and create her own genetic line, whilst her parents were not able to do this (the possible reason for adoption). Farnfield [28] found that almost a third of adoptive parents focus consciously on needing a baby with the implicit goal of creating a genetic line for the future. As this is not possible in the adoptive family, an internal conflict comes to the surface for the adoptive parents, meaning that they will often pull away from the attachment relationship they have built with their adoptive child as the adoptive parents try to understand how to navigate these feelings and thoughts. This situation can confuse the adoptive child, who may have previously felt loved and secure and is now being pushed away from the safety of their adoptive parents. The child will then act out in a way to try to gain that much needed and wanted attention back from their parents. In Joanna’s situation, her conflict is perhaps being shown through self-harm, questioning her gender and femininity, and looking for answers in her birth family. The family start a cycle, with everyone showing distress, rejection, and confusion. This can then lead to a previously stable placement breaking down, with the family and professionals not being able to articulate the exact reason.

The assessing therapist felt that by the end of the SFF, there were more questions than answers. A parallel process had been played out with neither family nor therapist being able to articulate where the critical cause lay. There was an intense need for safety for Joanna and this needed to be addressed first. The parents also needed further assessment so that they would feel safe enough to share those inhibited feelings and begin to explore a way to communicate as a family again.

**The Assessment of Parent–Child Interactions (ACPI):** To start to understand the underlying communication styles of the family the Assessment of Parent–Child Interactions [5] was undertaken with the full family to assess attachment behaviors and parenting capacity. The APCI is conducted face to face, over two 30-min sessions, held one week apart. The APCI contains four ‘musical activities’ which aim to highlight the nonverbal communication skills of the family, as well as levels of mutual attunement and parenting capacity. The four activities are Soft-Loud-Soft, Taking Turns, Follow my Leader, and a Free Improvisation.

The APCI, which was conducted with both parents over two sessions each, showed that there were some successful elements of the relationships between Joanna and Mr. Townsend and Joanna and Mrs. Townsend. The charts below detail the results for each area analyzed within the APCI (Figure A3 and Figure A4), the attachment behavior of the dyad (Figure A5 and Figure A6), and the parenting capacity of the dyad (Figure A7 and Figure A8).

Analysis of the interactions in the music therapy sessions showed that for Joanna and Mr. Townsend, their mutual attunement, nonverbal communication, and emotional parental responses were in the normal ranges. Joanna seemed to be more playful with Mr. Townsend than with Mrs. Townsend, although there was a sense that Mr. Townsend was mindful of Joanna’s physical vulnerability, which effected the level of playfulness and enjoyment in the session. For example, during the follow my leader activity, Joanna showed high energy levels and dynamic music playing. Mr. Townsend met her gaze and facial expressions well and although he started to meet Joanna’s musical energy too, he quickly diminished his efforts, in the possible hope that Joanna would follow and not become over excited.

With Joanna and Mrs. Townsend, their mutual attunement and nonverbal communication were both in the normal range, however, the emotional parental response was low. The assessing therapist felt that Mrs. Townsend did not always respond to the emotional needs of Joanna and there were some problematic behaviors, such as Mrs. Townsend turning away from Joanna with rejection and limited face to face contact. For example, when Joanna and Mrs. Townsend were participating in the taking turns activity, Joanne started playfully, with good eye contact towards Mrs. Townsend. Mrs. Townsend took a musical turn and then moved her body and turned her face away from Joanna when she could have reciprocated Joanna’s gaze and energy. Furthermore, during the free improvisation activity at the end of the APCI, Mrs. Townsend criticized Joanna’s music, saying that she could have done better, then she moved her chair away from Joanna.

Although it must be noted that Mr. Townsend’s score for emotional parental response (Figure A4) was in the normal range, it was still a low score. Both parents were dependent on support from the assessing therapist, which could indicate a feeling of uncomfortableness in the assessment sessions. Furthermore, the family seemed more comfortable in the structured activities, rather than the unstructured ones, again highlighting a need for supporting boundaries within the family.

Analysis of APCI Activities

Graphic representation of attachment behaviors

Graphic representation of parenting capacity

The output graphs for the APCI data were analyzed as per the APCI protocol (See Appendix B for more information). For Mr. Townsend, the analysis is based on Figure A6 and Figure A8 and indicates APCI profile 6—MCSI, which stands for Mutually Attuned, Clear Nonverbal Communication, Supportive Parenting and Independent Child. This APCI result is classed as ‘Good Enough Parenting”. For Mrs. Townsend and Joanna, Figure A5 and Figure A7, the analysis indicated APCI profile 7—MCLI, which means Mutually Attuned, Clear Nonverbal Communication, Lack of Parenting Support, and Independent Child. With this profile, the lack of parenting support, along with other positive elements, may confirm the findings within the Screening Family Formulation that the family are trying to be ‘good enough’ but there are mismatches in the relationship, potentially causing conflict. With the Townsend family, there was a possibility of the inhibition of the feelings between mother and daughter impacting negatively on their communication and functioning as a dyad.

**Interpretation and Evaluation of the SFF and the APCI with the family:** Using the SFF alongside the APCI could be seen to confirm, or at least provide focus on, the themes that are underlying the Townsend family’s difficulties. Throughout the SFF, there were questions around the ‘not knowing’ of reasons (or lack of negative events) which could have caused the crisis within the family. There was a feeling of something being hidden and without the knowledge from the DMM—i.e., looking for discrepancies in the reported information and the heightened thought on attachment strategies. This may have been colluded between the therapist and the family. The SFF had highlighted the potentially different attachment strategies being used by the family. Joanna was using a possible Type C3–4 strategies (shown in Figure 1 previously), oscillating between aggressive outbursts (self-harm) and helplessness (needing help with her somatic conditions). Her parents, conversely, could be seen to be using a Type A3–4, inhibiting negative affect. There was also some disorientation in the use of the strategies. As discussed earlier, disorientation is when individuals are not aware that their strategy is not functioning, and they become confused as they try to process information that feels mismatched to their feelings and behaviors. These thoughts were clarified with the addition of the APCI, where Joanna’s overt emotional requests were either ignored (during the APCI with Mrs. Townsend) or thought of as too overwhelming (with Mr. Townsend). Joanna’s feelings of not belonging and searching for an identity, although natural given her adolescent age and her experience of being adopted, were being magnified in the minimization of her feelings in the reflection of her parent’s feelings. Using the SFF’s ideas of creating questions to focus thought, Joanna might have been asking, “*If I have all of these feelings, and my parents don’t, then what is wrong with me*?”.

**Proposed Treatment Plans:** In conclusion, the SFF and APCI complimented the assessment for the family and brought greater depth in understanding for the family. Both assessment models offer the chance for professionals to use compassion by listening to the family, curiosity when formulating questions in the SFF and noticing behaviors in the APCI and also holding hope for the family through an individualized treatment plan for the future. After the assessment was completed, and alongside the medical interventions which were necessary for Joanna, two treatment pathways were offered to the family. As the SFF had highlighted a disorientation of attachment strategies alongside gaps in information and the potential for crisis to happen, a Full Family Formulation was offered with an Adult Attachment Interview for parents and a Transition to Adulthood Interview for Joanna. The family were showing signs of low resilience and awareness, and so to support this in the more immediate future, the family were also offered art therapy for Joanna [30] and psychological therapy for her parents. The hope for the future of the family was to bring together their opposing attachment strategies, create a place for emotions and experiences to be talked about with less shame, and to build positive feelings within the home. The Gradient of Interventions from the SFF (Figure A9) was used to help decide the treatment plan, and the choice for which to pursue was offered to the family.

## Appendix B. APCI Graphs Information

The APCI proposes family profiles based on the results shown in the graphs, highlighted above. The information on mutual attunement is taken from activities one and three (Soft-Loud-Soft, Follow my Leader), nonverbal communication data is gathered from activity two (Taking Turns) and the emotional parental response data is gathered from activities one, three, and four (Soft-Loud-Soft, Follow my Leader, Free Improvisation). Analyzing the number of following and leading events from both child and parent, the information is added to a database which collates the overall scores for the family. This resulting information is shown in graphic form (Figure A5, Figure A6, Figure A7 and Figure A8). The two topics of graphs, Attunement and Emotional Support, highlight the attachment behaviors presented by the family as well as the potential emotional support experienced by parent and child. This is then linked to the APCI family profiles, which provide the assessing therapist with information on how the family are functioning together. There are 16 profiles in total, with profiles 1–6 showing ‘good enough parenting, profiles 7–13 highlighting ‘at risk’ families, and the final four profiles indicate possible neglect and/or abuse in the home.
